# Suicide as a public health concern: Confronting the means, motives, and opportunity

**DOI:** 10.1093/eurpub/ckac131.471

**Published:** 2022-10-25

**Authors:** J Overholser, C McGovern, C Silva, S Gomez

**Affiliations:** Psychology, Case Western Reserve University, Cleveland, USA

## Abstract

**Issue:**

Suicide continues to present a major public health concern in many societies. Suicide prevention efforts have failed to reduce the rate of suicide in most countries. A change of intervention strategy can help to prevent these deaths from despair.

**Description of the problem:**

Suicide prevention strategies are examined through an integrative review combined with more than 30 years of experience conducting research on suicide attempters and psychological autopsy research on adults who had died by suicide. Journal articles published since 2005 were reviewed for strategies designed to prevent death by suicide.

**Results:**

Suicide risk is examined by confronting the means, motives and opportunities for suicidal behavior. Prevention strategies that limit access to lethal means can have a beneficial impact if the method is easily accessible and not easily replaced. Unfortunately, some individuals merely shift to a different method for their suicidal act. Prevention strategies that restrict the opportunity for self-injury provide time to confront underlying disorders and initiate treatment. Unfortunately, many patients do not continue treatment beyond the acute crisis, and there is often a resurgence of suicidal behavior after discharge from the hospital. Prevention strategies that aim to confront the person’s motivation to die may reduce the underlying cause. It is important to provide interventions to help reduce the desperation and isolation that underlie suicidal urges. These intervention strategies hold potential for making lasting changes that could eliminate, instead of temporarily suppressing, the desire to die.

**Lessons:**

Restricting access to lethal methods and limiting times when a suicidal person is left alone can temporarily block suicidal urges. However, the motive underlying the suicidal urges remains intact. The motivation to die can be addressed through interventions that focuses on helping clients to build a life worth living.

**Key messages:**

• Suicide prevention strategies can confront the desire for death by helping to build a life that is worth living.

• Comprehensive interventions aimed at reducing depression, isolation and addiction hold potential for reducing the rate of suicide.

